# Access to health knowledge for health equality: a multi-phase review focused on disability-health

**DOI:** 10.1186/s12939-023-02080-5

**Published:** 2024-01-10

**Authors:** John C. H. Hu

**Affiliations:** 1 University of Alberta, 3-58 Corbett (E.A.) Hall, 8205 - 114 St NW, Edmonton, AB T6G 2G4 Canada; 2 University of Manitoba, Winnipeg, Canada; 3 Simon Fraser University, Burnaby, Canada

**Keywords:** Inequality, Accessibility, Communication, Information, Research dissemination, Social determinants of health, Knowledge translation, Knowledge mobilization, Knowledge transfer

## Abstract

**Background:**

The existing evidence base indicates increased interest in knowledge translation (KT), or, the dissemination of research to ensure uptake and impact. Given this definition, this study aimed to review existing scholarship on knowledge translation (KT) of health research to people living with disabilities (PLWD), and assess the current state of accessibility of health knowledge for people living with disabilities.

**Methods:**

Given existing heterogeneity in literature as well as a number of varying definitions for both disability and knowledge translation, a reflexive, three-phase approach was utilized to improve methodological soundness. Phase I recognizes that existing review-style studies have been conducted on disability-KT. An existing systematic review on KT specific to the field of rehabilitation and physical medicine was analyzed to assess potential best practices towards inclusivity and accessibility for people living with disability. Phase II used the *Center on Knowledge Translation for Disability and Rehabilitation Research (KTDRR)* database as an information-source with high-specificity to disability-health KT. Phase III sought to rapidly assess the current landscape of systematic reviews relevant to disability-health KT, with four systematic reviews meeting the inclusion criteria across Cochrane, Psycinfo, CINAHL, PubMed, Web of Science, and EMBASE.

**Results:**

The current landscape of disability-health KT is primarily targeted at health professionals who serve PLWD. PLWD are included in KT, mostly as key informants, or as study participants in KT-studies designed as health interventions. Multiple systematic reviews on disability-health KT exist, presenting vastly different foci which prevent assessment of best practices.

**Conclusions:**

KT efforts are abundant and can be seen across health research related to disabilities, generating considerable literature and systematic reviews. With regards to meeting the public health objective of equalizing and enhancing access to health knowledge, future knowledge translation efforts intending to provide PLWD with up-to-date health research can be of significant value.

## Key messages

- *Knowledge translation* emerges as the most prominently used terminology to describe research dissemination; yet, its use in a search strategy often identifies studies in which people living with disabilities as sources of knowledge as opposed to recipients of knowledge

- Intentional strategies to provide equitable access to health information for people living with disabilities can be multi-phase and highly complex if the study is not structured as a health intervention

- The existing landscape of systematic review on disability-KT may emphasize evaluation of the effectiveness of KT; future studies on best practices for equitable access to health research can be of significant value

## Introduction

People living with disabilities (PLWD) may face exclusion or barriers in accessing information, including research-based information about disabilities. ###Public health is increasingly aware of the significance of disseminating research-based information [1], which is corroborated by the proliferation of academic language to describe this concept. In the English language alone, it is estimated that over one-hundred terms are available to describe the process of research dissemination [2]. Terminology such as *knowledge translation* [3], *knowledge mobilization* [4], and *knowledge transfer* or *knowledge-to-action* [5] find varying degrees of use within health-equity research. For the purposes of this study, knowledge translation (KT) will be the term of choice for brevity.

Knowledge translation of health research may be considered primarily as providing information to health professionals as opposed to patients, as seen in various KT studies to be described in the Results section. At the same time, knowledge translation has also been considered as specifically inclusive of and tailored to lay participants - including notable, integrated attempts such as the Cochrane Consumer Network. This distinction is further complicated by the precise information to be translated or communicated. For example, information contributing to basic health literacy may be conceptualized as distinct from information involving the most up-to-date research findings disseminated under funder requirements. A third category of information could be conceptualized as lived experience with a disability and lived experience of managing a disability - both of which may involve information which are not yet fully captured in recent research. All three categories of information are valid for PLWD to have access to, as the knowledge combined can present potential to support equitable health outcomes. For the purposes of this study, the following considerations on positionality are included below to provide scope on what is meant by *knowledge translation*. The fact that research is being produced and disseminated about PLWD suggests that PLWD should be able to access what is being disseminated about them and their disabilities if they choose to participate in this public discourse. Secondly, what is conceptualized as research outputs intended only for policymakers, occupational therapists, physicians, and other stakeholders could also benefit from KT that is inclusive to PLWD, as PLWD may in turn become supported into roles as future policy makers and health professionals. As such, the study takes the position that improving PLWD’s access to all forms of research information may be beneficial, and that each individual PLWD can benefit from autonomy to determine what is valuable information to access. KT practices which offer PLWD inclusive access to research information - intended for PLWD or not - may therefore be considered as valuable, particularly from a perspective of supporting the reduction of health and social inequalities experienced by this population. For brevity, knowledge translation which supports equitable access to health information for PLWD are noted hereon as disability-KT.

Traditionally, knowledge translation outputs under the requirements of funders mainly take the form of peer-reviewed articles or academic conferences, both of which can be associated with formal academic opportunities. For PLWD, access to formal education is often inequitable around the world [6] which may further inhibit access to the traditional outputs of knowledge translation. These gaps in knowledge translation extend beyond an issue of equitable access, but may have implications for efficiency in public health: currently, it is estimated that lack of knowledge translation leads to a global waste of $760 to $935 billion USD dollars per year in research funding [7]. For individuals requiring health services, the lack of knowledge translation is also estimated to result in up to 45% of patients receiving inadequate care, and an additional 25% of patients receiving unnecessary or harmful care [8] - ultimately leading to a noteworthy health concern of population scale.

## Methods

The objectives of the study are: 1) to understand the current state of access-to-knowledge for PLWD specifically regarding health research, and 2) to compile and provide an overview of KT best-practices and KT tools employed by existing studies which engage PLWD in disability-health knowledge translation.

This study, following the basic structure of a narrative review, applies a reflexive research design [9] in recognition of two important sources of heterogeneity in the literature: first, knowledge translation can be described by a multitude of terms, and second, various research studies emphasize a single disability or impairments in function, suggesting that rigor in assessing universality of data will require significant dedicated intent. A multi-phase structure was adapted as follows.

### Phase I

The first, exploratory phase of the study recognizes existing review-style studies exist on disability-KT, and identifies a review meeting the objectives of this paper. Moore et al. [10] applied a broad definition of disabilities, and assessed 3120 knowledge translation and knowledge-to-action articles directly related to *rehabilitation medicine* and *physical medicine* (ie. physical therapy). Phase I involved full-text analysis of the 46 articles representing 37 unique studies meeting the inclusion criteria of Moore et al., in order to assess current state of access-to-knowledge for PLWD. Results informed the design of Phase II, as Moore et al. presents 9 studies improving access to knowledge for PWLD with 26 improving access for health professionals serving PLWD.

### Phase II

The proportion of articles relevant to improving access-to-information for PLWD in Phase I informed the direction of the second phase. Given the fact that other systematic reviews on disability-health KT may also merge KT targeted at health professionals together with KT targeted at PLWD, an intentional decision was made to identify information sources specific to improving access-to-knowledge for PLWD. A search eventually identified the *Center on Knowledge Translation for Disability and Rehabilitation Research* as a high-specificity information source towards understanding the existing landscape of disability-health KT. The mission of the Center on Knowledge Translation for Disability and Rehabilitation Research (KTDRR) is “to make it easier to find, understand, and use the results of research that can make a positive impact on the lives of people with disabilities” [11]. The website includes a searchable Knowledge Translation Strategies Database, which as of April 1st, 2023 provides access to 217 sources on disability-health KT. The database is searchable by 11 target audience groups, which are divided into the following: *Administrator; Business/Employer/Industry; Decision Maker; Educator; Healthcare Professional; Person with disability/Family/Advocate; Policymaker/Legislator; Research Funders; Researchers; Service Provider;* and *VR Professional.* Twelve studies were identified through searching for KT strategies with *Person with disability/Family/Advocate* as the target group; these 12 studies were screened for peer-reviewed nature. Seven peer-reviewed studies emphasize PLWDs engaged as recipients-of-information.

### Phase III

Phase II demonstrated that among 217 sources of disability-health KT, seven sources representing 3.2% of the database emphasized increasing access to health-research for PLWD. In an attempt to identify more conglomerate sources of information on disability-health KT, Phase III was strategized as a rapid search for existing systematic reviews on disability-health KT. Phase III was initiated with a search in the following databases: Cochrane, PsycInfo, CINAHL, PubMed, Web of Science, and EMBASE, retrieving systematic reviews in the English language from 1970 to April 1st, 2023 (Table 1).

**Table 1:** Search strategy for phase III, preliminary assessment of systematic reviews

**Field(s)**	**Term(s)**
Title	“knowledge translation” OR “knowledge mobilization” OR “knowledge transfer” OR “knowledge exchange” OR “knowledge dissemination” OR “research dissemination”
Title	“systematic review”
Title/Abstract/Keyword	disab* OR rehab* OR physiothera* OR “occupation therap*” OR “physical medicine”

Data extraction was completed via analyzing all full texts for the following. First, *target group*; studies which engaged PLWD as the only or part of a larger target group for receiving health knowledge were all included. Secondly, *best practices*, which constitute principles or study designs employed towards disability-health KT. Third, *terminology* used to describe the KT process as employed by the study authors. Fourth, *tools*, which are more concrete in nature and may be used in conjunction with best practice principles.

## Results

### Phase I

Moore et al. filtered 3120 articles to 46 by the author’s emphasis on understanding knowledge-to-action frameworks applied in disability-health research. These 46 papers include 11 studies engaging PLWD as part of the target audience for KT. These 11 studies were reviewed to gather insights on improving PLWD’s access to health research; the data was filtered down to 9 unique projects which fully met the objective of this study. Table 2 outlines the best practices, target group, terms, and tools used by each of the 9 unique projects involving PLWD in health-KT.

**Table 2:** Phase I studies which met inclusion criteria of involving PLWD in disability KT

**Project**	**Target Group**	**Best Practice**	**Term**	**Tools**
Brosseau et al. 2012 [12]	Individuals with mild to moderate knee osteoarthritis	Single-blind randomized control trial	KT *and* KTA	Supervised community walking program, and educational pamphlet
Dew and Boydell, 2017 [13]	Providers and users of rural therapy services in Australia	Partnership between PLWD, their support networks, and practitioners	KT *and* KTA	Survey results converted into infographic
Li et al. 2013 [14]	Individuals with early osteoarthritis	Single-blind randomized control trial and behavioral theory	KT *and* KTA	Behavioral theory-informed Internet intervention and educational pamphlet
Liu et al. 2013 [15]	Patients > 65 years old, admitted to acute or continuing care hospitals	Site-tailored analysis of barriers and facilitators to research uptake by PLWD	KT *and* KTA	Local champions, online and/or in-person educational interventions for healthcare providers and patients, printed education materials, implementation coaching, and an online community of practice
Munce et al. 2014 [16]	Individuals with spinal cord injuries (*n* = 7); family members; rehab professionals	Understand PLWD’s perspectives on barriers and facilitators to self-management, specifically impact of mood and outlook	KT *and* KTA	Semi-structured telephone interviews
Novak, 2014 [17]	Parents of children with cerebral palsy	Tailor KT to questions that parents ask neurologist about their child	KT *and* KTA	Social media survey to engage parents
Rafferty et al. 2019 [18]	Individuals with early stage Parkinson’s Disease	Acknowledge barriers of location; insurance coverage; and challenge associated with scheduling long-term follow-up visits	KT *and* KTA	Intervention program involving regular exercise
Tsui et al. 2015 [19]	Community dwelling seniors post- hip fracture; family members	Allowing PLWD to rate various aspects of the design of a KT tool	KT *and* KTA	Education manual
Tugwell et al. 2007 [20]	“Consumers”	Research-intensive approach combining surveys, semi-structured interviews, and development of evaluation metric	KT *and* KTA	Consumer summaries

Phase I illustrates that PLWDs can be largely engaged as study participants with KT projects structured as health-interventions. Out of the 9 unique projects, 4 were designed as health interventions for PLWDs [12, 14, 15, 18].Out of these 4 projects, 3 are based on interventions to promote physical health [12, 15, 18] with 1 evaluating a website as a health-intervention for PLWD [14].

Beyond projects which emphasized experimental design of a health intervention as KT, an additional 4 projects engaged PLWDs to learn their perspectives for the purpose of informing researchers or practitioners [13, 16, 17, 19]. In totality, Phase I presents 8 out of 9 projects engaging PLWDs either as study participants, or as informers to enhance knowledge of health professionals.

One remaining project from Phase I emphasized improving access to research for PLWD. The study serves to illustrate the massively-complex nature of KT that is aimed at improving PLWD’s access to knowledge: Tugwell et al. [20] sought to improve PLWD’s access to research outputs via creation of tailored “consumer summaries”. In the context of the study, Tugwell et al. defines “consumers” as individuals who used services from Arthritis Society of Canada, Canadian Arthritis Patient Alliance, and the Cochrane Consumer Network. The authors engaged one-hundred and ten consumers through a web survey to identify the types of information which were of interest to PLWD, and then conducted a review of both literature and existing patient information guidelines to meet the specific requests of PLWD. Resulting data was tailored into one-page, plain language summaries with highlights for ease of information-access.

Tugwell et al.’s KT process demonstrates significant overlap between KT and the health research process itself. After the authors’ initial survey involving 110 respondents, 78 people with disabilities were engaged in a follow-up phase of semi-structured interviews. The qualitative data was used to understand the information-needs of PLWD, as well as their perceived gaps in disability-KT.

Tugwell et al.’s KT process continued beyond the semi-structured interviews. Researchers then considered barriers and facilitators in disability-KT, and applied a public-health lens focused on “broad dissemination” of their consumer summaries towards equitable reach. Evaluation of effective KT concluded Tugwell et al.’s comprehensive project methodology, which involved the development of a measurement instrument - the Effective Consumer Scale - for monitoring the effectiveness of sustained KT.

Tugwell et al. illustrates that improving access to knowledge for PLWD may involve massively-complex, multi-phase processes. KT that is tailored towards PLWD’s equitable access to knowledge, therefore, may resemble research in itself rather than a post-research dissemination project. The complexity and effort required to tailor accessible health information for PLWD may contribute to lack of similar studies on disability-health KT. Overall, Phase I illustrates that PLWD are currently largely engaged as informers of knowledge or study participants in disability health interventions, with Tugwell et al. being a study emphasizing access to knowledge for PLWD.

### Phase II

Out of the 217 sources available in the Center on Knowledge Translation for Disability and Rehabilitation Research (KTDRR) database, 12 can be retrieved through selecting PLWD as a target audience through a search-function available in the database design. All 12 studies were reviewed for their full text. One study did not meet the criteria of being a peer-reviewed article; 3 studies did not meet the criteria of involving PLWD in the KT process. In total, Phase II presents 7 studies which were peer-reviewed and involving PLWD as a target group for KT.

Prior to the beginning of Phase II, it was anticipated that new terms used to describe KT in the disability-health fields would be discovered. One out of 7 studies in Phase II used knowledge-to-action (KTA) as the preferred terminology to refer to KT [21]. All other studies used KT, with one study using KT in combination with two new terms: *knowledge adoption* and *knowledge scale*-*up* [22]. In combination with Phase I, existing literature would suggest prominent usage of the term KT to describe the research dissemination process in health research. Table 3 outlines the 7 studies by *target group, best practices,* and *tools* used for disability-health KT.

**Table 3:** Phase II studies which met inclusion criteria of involving PLWD in disability KT

**Project**	**Target Group**	**Best Practice**	**Term**	**Tools**
Anaby et al. 2021 [21]	Paediatric rehab professionals; patients and families	Five-way partnership of “family-clinician-Manager-community leader-policymaker”; creation of a KT roadmap	KTA	Decision aids and logic models
Cross et al. 2018 [23]	Children with disabilities	5 F’s: function, family, fitness, fun, friends, and future	KT	Evaluation instrument
Vanderbom et al. 2018 [22]	General population	A KT Centre: “adapting knowledge, facilitating uptake, developing strategic partnerships, and building community capacity”	KT, K adoption, *and* K scale-up	Online resources survey and web-based training
Reimer-Kirkham & Jule 2015 [24]	General population	“Crosstalks” between patients and service professionals	KT	Knowledge cafe
Hall et al. 2014 [25]	Intellectual disabilities	Family involvement	KT	Social network analysis
Kastner et al. 2014 [26]	Individuals at risk for osteoporosis	“Patient-initiated risk assessment questionnaire (RAQ)”	KT	“Multifaceted Interventions”
Shooshtariet al. 2018 [27]	Intellectual and developmental disabilities	Overcoming challenges with “relationships and interaction between parents and practitioners as facilitators and lack of resources, time and incentives”	KT	Focus group and stakeholder-workshops

Phase II illustrates significant heterogeneity among existing literature based on target group. Three out of 7 studies include PLWD, but do not have PLWD as the primary target group: out of these 3 studies, 2 studies discuss disability-KT to the general population from a public health perspective [22, 24], and 1 study engaged individuals who are at risk of osteoporosis but may or may not live with a disability [26]. This would be the first study to emerge with a preventative lens on disability. Notably, in comparison to Phase I, Phase II presents no KT studies which are structured as health-interventions. This suggests that analysis of the different existing conglomerate sources of disability-KT literature may lead to considerably varying conclusions.

Various best practices and tools emerge in performing Phase II of the study. Given existing heterogeneity, a framework to assess applicability of these practices and tools across different disabilities, study-foci, and geographic context is not readily available. As such, these best practices and tools for disability-specific KT are identified in Table 3 only to facilitate future investigations on how the existing evidence can be best translated in new KT projects. In combination, the suggested best practices and tools present significant diversity and do not necessarily overlap with the KT-practices identified in Phase I. This diversity was used to inform the development of Phase III.

### Phase III

Due to the diverging themes from Phase I studies and Phase II data analysis, it became apparent that there is potential value in conducting a rapid overview of existing conglomerate sources on disability-KT. The following search strategy was designed to retrieve systematic reviews related to health-KT involving PLWD in Cochrane, PsycInfo, CINAHL, PubMed, Web of Science, and EMBASE. The search strategy for Phase III is outlined in Table 1. Out of the six databases, 17 out of 31 systematic reviews retrieved through the search strategy were duplicates. None of the systematic reviews retrieved in Phase III were captured in Phase I or Phase II. For the 14 unique studies, screening for inclusion criteria resulted in the following flow-chart (Table 4).

**Table 4:**
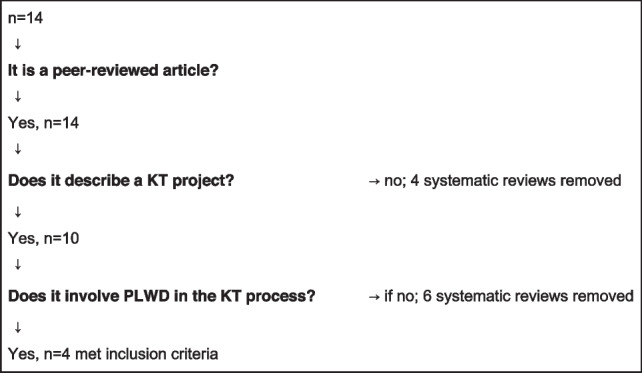
Phase III flow chart for screening of systematic reviews

Data analysis of Phase III reinforced notions of heterogeneity within the existing evidence base. Although various systematic reviews relevant to disability KT can be retrieved, existing literature is organized around different health conditions, as well as different research objectives. Table 5 organizes the 4 studies by providing overview on: 1) the databases used in each systematic review and number of studies meeting the authoring team’s inclusion criteria; 2) the authoring team’s objective in conducting the systematic review; and 3) results drawn by each systematic review.

**Table 5:** Phase III systematic reviews which met inclusion criteria of involving PLWD in disability KT

**Title of study**	**Database & number of articles**	**Objective**	**Results**
*A Cochrane review of patient education for neck pain* [28]	Index to Chiropractic Literature, MANTIS; MEDLINE, EMBASE, CENTRAL, CINAHL, CENTRAL, screening of references, communication with the Cochrane Back Group, and content experts (*n* = 15)	Evaluating effectiveness of KT for patients	“No effectiveness for educational interventions, including advice to activate, advice on stress-coping skills, workplace ergonomics and self‐care strategies” [28]
*Systematic review of knowledge translation strategies in the allied health professions* [29]	MEDLINE, CINAHL, ERIC, PASCAL, EMBASE, IPA, Scopus, CENTRAL (*n* = 32)	Categorizing types of KT tools and outcomes of KT	KT tool: educational meetings (*n* = 11), educational materials (*n* = 2), educational outreach visits (*n* = 1), and a financial intervention (*n* = 1, for KT related to HIV pharmaceuticals) KT outcomes: professional/process outcomes (*n* = 25); patient outcomes (*n* = 4), economic outcomes (*n* = 2), “multiple primary outcomes” (*n* = 1)
*Exploring the function and effectiveness of knowledge brokers as facilitators of knowledge translation* [30]	MEDLINE, Embase, PsycINFO, CINAHL, ERIC, Scopus, SocINDEX, and Health Business Elite (*n* = 29)	Summarizing list of all tasks completed by knowledge-brokers in KT	“Identify, engage, and connect stakeholders; Facilitate collaboration; Identify and obtain relevant information; Facilitate development of analytic and interpretive skills; Create tailored knowledge products; Project coordination; Support communication and information sharing; Network development, maintenance, and facilitation; Facilitate and evaluate change; Support sustainability”
*A systematic review of the effectiveness of knowledge translation interventions for chronic noncancer pain management *[31]	Cochrane, MEDLINE, EMBASE, Centre for Reviews and Dissemination Database, CINAHL, Web of Science, PsycINFO, Sociological Abstracts, SocINDEX, Social Services Abstracts, ABI Inform, Business Source Complete, Health-evidence.ca, KT+, Campbell Collaboration, Knowledge Utilization – Utilisation des Connaissances, Canadian Research Index (*n* = 19)	Review of targets and tools of KT	*n* = 13 to practitioner KT *n* = 4 to patient KT *n* = 2 to combined KT **Tools for patient KT**: information/education, decision aids, coordination-of-care **Tools for combined KT**: educational approaches, mass media**,** health promotion

Phase III illustrates that potential umbrella reviews on disability-KT can face challenges of both limited data as well as new sources of data heterogeneity uncovered in Phase I and Phase II. Scott et al. considered disability-KT, but also included non-disability KT in their study as the authors combined KT in occupational therapy, physiotherapy, speech-language pathology, but also in dietetics and pharmacy in the same systematic review [29]. This is in contrast to systematic reviews with extremely refined focus, for example, Haines et al. which only emphasized only neck pain [28] and concluded that there is no effectiveness of education-based KT on improving health outcomes.

Notably, 2 out of 4 systematic reviews retrieved by the rapid search strategy focus on pain. Ospina et al. did not draw the same conclusion as Haines et al. on lack of effectiveness of education-based KT, but rather concluded that no singular KT strategy could be recommended to be optimal in all contexts [31]. Ospina et al. also corroborates findings from the previous two phases: out of the 19 articles meeting their inclusion criteria, 15 involved KT to health professionals.

Bornbaum et al. did not emphasize disabilities, but considered disability-KT as part of a larger discussion on the role of “knowledge brokers” in the KT process. Like Haines et al., the authors state no definitive conclusions can be drawn on the effectiveness of involving knowledge-brokers in KT. In totality, Phase III illustrates that further investigation into conglomerate sources on disability-KT will possibly engage with content regarding non-disabled patients. Phase III would also suggest that existing conglomerate sources lean not towards identifying best practices or tools, but rather on evaluating *effectiveness* of KT with few positive conclusions. Among the four disability-KT systematic reviews retrieved in Phase III, three would comment on inconsistent methodologies and low-quality of evidence as hindrance to drawing definitive conclusions after the review process.

## Conclusions

A range of conglomerate sources exist on disability KT, suggesting considerable interest in its academic investigation. Out of the three phases of this study, Tugwell et al. presents an academic endeavor in which PLWD are intentionally engaged as recipients of knowledge in health research. Notably, their study is multi-phase and complex in nature, suggesting that intentional efforts to improve access-to-research for people living with disabilities can involve unconventional KT-designs. Another key finding which emerges from analyzing Tugwell et al. is that the paper employs terminology which may be difficult to capture in conventional search strategies. The study is the only one among three Phases which uses consumers to describe PLWD, and thus leads to KT-outputs being described as consumer summaries. This suggests that while KT is prominently used to describe research-dissemination efforts in existing health research, other innovative terminologies can exist to retrieve highly-relevant evidence in the literature.

Recurring themes across the three phases is that disability-KT is currently more often targeted at health professionals as opposed to PLWD. In assessing KT studies involving PLWD, access-to-knowledge is not always part of study objectives. PLWD are largely involved as informers to health professionals, suggesting that the need to amplify the voices of the marginalized may be prioritized over access-to-knowledge efforts for the same marginalized population. PLWD are also engaged as study participants in KT studies which are structured as health interventions. Using the example of a walking-intervention from Phase I [12], it can be assumed that the PLWDs engaged in a health-promoting KT intervention will gain greater knowledge on the benefits of walking as physical activity. At the same time, the total numbers of PLWD engaged through KT structured as health interventions may be of interest when applying a population-health lens.

Overall, the term KT has utility in capturing a large number of studies around disseminating health knowledge to the public. However, the conceptualization of KT as an two-directional process - in which voices of the community are also shared to the health professionals - may lead to studies which do not emphasize access-to-knowledge for PLWD. The current conceptualization of KT as part of the implementation science umbrella may also lend itself to studies involving health-intervention design, in which PLWD are engaged as study participants and not necessarily recipients of health knowledge.

This study corroborates previous findings on KT discourse in public health: knowledge translation and other similar terminology are numerous, varying, overlapping, and thus are “seen as themselves requiring translation” [32]. Still, the state of PLWD as a marginalized population who already experience negative health outcomes suggests value in intently improving their access to health knowledge. Successfully achieving this objective may require multi-phase efforts which commit to not just disseminating research, but also engaging PLWD to understand their information needs; tailoring information in their desired forms; and evaluating the KT process based on a PLWD-centered qualitative inquiry. A dichotomy thus emerges in which a simplified conceptualization of KT may help clarify what exactly is knowledge translation, but effective KT to marginalized populations may be inherently complex in nature in contrast. The case of KT in disability-health suggests that improving access-to-knowledge for people living with disabilities remains a valid effort amidst the multitude of disability-KT projects.

## Data Availability

The datasets used and/or analysed during the current study are available from the corresponding author on reasonable request.

## Abbreviations

KT: Knowledge translation
PLWD: People living with disabilities
